# Efficient sparse-view medical image classification for low radiation and rapid COVID-19 diagnosis

**DOI:** 10.1007/s13534-025-00478-4

**Published:** 2025-05-22

**Authors:** Seunghyun Gwak, Sooyoung Yang, Heawon Jeong, Junhu Park, Myungjoo Kang

**Affiliations:** 1 Computational Science & Technology, Seoul National University, 1 Gwanak-ro, Seoul, Gwanak-gu 08826 South Korea; 2 Interdisciplinary Program in Artificial Intelligence, Seoul National University, 1 Gwanak-ro, Seoul, Gwanak-gu 08826 South Korea; 3 Department of Mathematical Sciences and Research Institute of Mathmatics, Seoul National University, 1 Gwanak-ro, Seoul, Gwanak-gu 08826 South Korea

**Keywords:** Computed tomography, Image classificiation, COVID-19 diagnosis, Sparse-view sinogram, Masked autoencoder

## Abstract

This study proposes a deep learning-based diagnostic model called the Projection-wise Masked Autoencoder (ProMAE) for rapid and accurate COVID-19 diagnosis using sparse-view CT images. ProMAE employs a column-wise masking strategy during pre-training to effectively learn critical diagnostic features from sinograms, even under extremely sparse conditions. The trained ProMAE can directly classify sparse-view sinograms without requiring CT image reconstruction. Experiments on sparse-view data with 50%, 75%, 85%, 95%, and 99% sparsity show that ProMAE achieves a diagnostic accuracy of over 95% at all sparsity levels and, in particular, outperforms ResNet, ConvNeXt, and conventional MAE models in COVID-19 diagnosis in environments with 85% or higher sparsity. This capability is especially advantageous for the development of portable and flexible imaging systems during large-scale outbreaks such as COVID-19, as it ensures accurate diagnosis while minimizing radiation exposure, making it a vital tool in resource-limited and high-demand settings.

## Introduction

Advancements in X-ray and CT imaging have revolutionized disease diagnosis by enabling non-invasive detection of internal organ abnormalities. In particular, during the COVID-19 pandemic, chest CT scans became indispensable due to their superior accuracy in diagnosing and assessing disease severity compared to conventional X-rays [18, 38]. However, acquiring high-quality CT images requires substantial radiation exposure and time-consuming image processing, which increases both risk and delays.

Sparse-view CT imaging has emerged as a promising solution to reduce radiation exposure by minimizing the number of X-ray projections [12, 13]. Prior works in sparse-view CT reconstruction have explored various approaches: CNN-based low-dose CT (LDCT) methods map sparse-view images to high-quality images yet often struggle with diverse training data [11], while GAN-based methods can improve artifact suppression, but face problems with training instability [12]. Other studies have proposed dual-domain methods that combine sinogram and image-domain processing to enhance artifact reduction and detail accuracy [13], though these techniques generally involve complex training and high computational costs.

Inspired by masked language models like BERT [14], recent developments in masked image modeling (MIM) have led to methods such as MAE and I-JEPA [15], which reconstruct masked regions in images to learn robust features. However, these approaches often overlook the specialized needs of medical imaging, particularly for sparse-view scenarios. Our work adapts the MIM framework to the unique properties of sinograms and implements a column-wise masking strategy to leverage the characteristics of sparse views, thereby eliminating the need for CT image reconstruction and enhancing diagnostic accuracy.

This study presents the Projection-wise Masked Autoencoder (ProMAE), a deep learning model designed for the direct diagnosis of COVID-19 and pneumonia from random sparse-view X-ray sinograms. Built on a Vision Transformer (ViT)-based Masked Autoencoder [9], ProMAE employs a sinogram-specific masking strategy to achieve over 97% classification accuracy with as few as 5% of the projections, without requiring reconstruction or post-processing. Furthermore, by robustly learning classification features from a small, randomly sampled subset of projections, ProMAE can deliver reliable diagnostic results across diverse acquisition environments and imaging outcomes.

The key contributions of this study are as follows.

1. In the ProMAE pre-training process, a tokenization and masking strategy suited for random sparse-view sinograms is applied. This enables the model to robustly learn the pathological features of COVID-19 across various levels of sparsity (50%, 75%, 85%, 95%, and 99%).
2. In the downstream task, the same masking strategy used during pre-training was employed to train a classifier that directly categorizes random sparse-view sinograms based on the extracted features.
3. Through pre-training and downstream tasks, ProMAE achieved a classification accuracy of over 95% on sparse-view data with 85% or higher sparsity, without requiring CT image reconstruction.

By processing sinograms directly and applying column-wise masking, ProMAE not only eliminates the need for CT image reconstruction but also reduces the reliance on dense projection data and high-performance hardware. This approach minimizes radiation exposure and paves the way for the development of lightweight and portable imaging devices, an especially valuable advance during large-scale outbreaks such as COVID-19.

## Datasets and methods

### Datasets

We use a lung CT dataset for COVID-19 screening, compiled from seven publicly available sources [16, 17, 33–37]. CT imaging, shown to outperform chest X-rays (CXR) in diagnostic accuracy [48], is recommended for initial assessments when feasible, considering capacity and radiation exposure [18].

**Table 1:** Dataset split for 3 labels

${\text{Labels}}^{1}$	Original set	Used dataset	Training	Test
NonCOVID	6,893	6,893	6,205	688
COVID	7,593	6,617	5,942	675
CAP	2,618	2,618	2,357	261
Total	17,104	16,128	14,504	1,624
			(89.93%)	(10.07%)

^1^NonCOVID refers to normal patients, COVID refers to patients infected with COVID-19, and CAP refers to patients with Community-Acquired Pneumonia

The dataset categorizes CT slices into NonCOVID (normal), COVID (infected), and CAP (community-acquired pneumonia). After excluding 976 low-quality images, a total of 16,128 CT slices remain, which are divided into training and validation subsets (Table 1). To mitigate structural biases arising from various acquisition environments, the $512\times 512$ CT images are resized to $256\times 256$, and random zooming ($1.2\times -1.5\times$) is applied to generate augmented ground truth (GT) images. Since the original sinogram data used for reconstructing the CT data in this study was not available, sinogram data had to be obtained via forward projection from the CT dataset [12, 23]. When the acquired sinogram was reconstructed into CT images, it exhibited a high average similarity to the original CT images (ssim: 0.8489), confirming that it effectively preserves the original details. The GT CT images are converted into GT sinograms, which consist of 360 projections ($256\times 360$) acquired at $0.5^{\circ }$ intervals. By removing $r\%$ of the projections from the GT sinogram and reconstructing the images using filtered back projection (FBP), sparse-view datasets are created with sparsity levels of 50%, 75%, 85%, and 95%. These datasets maintain the same structure as the GT CT dataset.

**Fig. 1:**
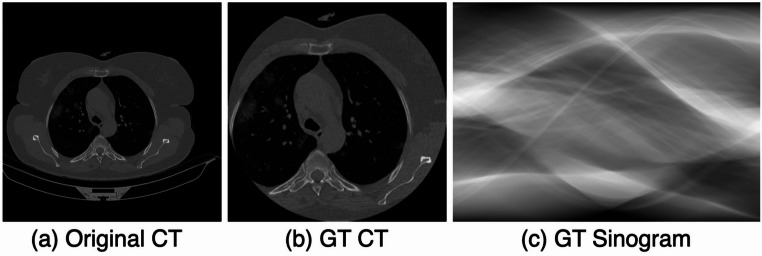
Pre-processing CT Images: **a** Original CT image, **b** Ground Truth CT image with biased information removed, and **c** its transformation into a sinogram

### Masking strategy on sinogram

ProMAE processes sinograms instead of CT images, thereby eliminating the reconstruction step. Each column of a sinogram corresponds to a projection at a specific angle, and each row represents a combination of sine functions. Unlike MAE models that tokenize square-shaped patches (e.g., $16\times 16$), ProMAE employs column-wise tokenization to preserve projection information, enabling effective learning even under high masking ratios.

**Fig. 2:**
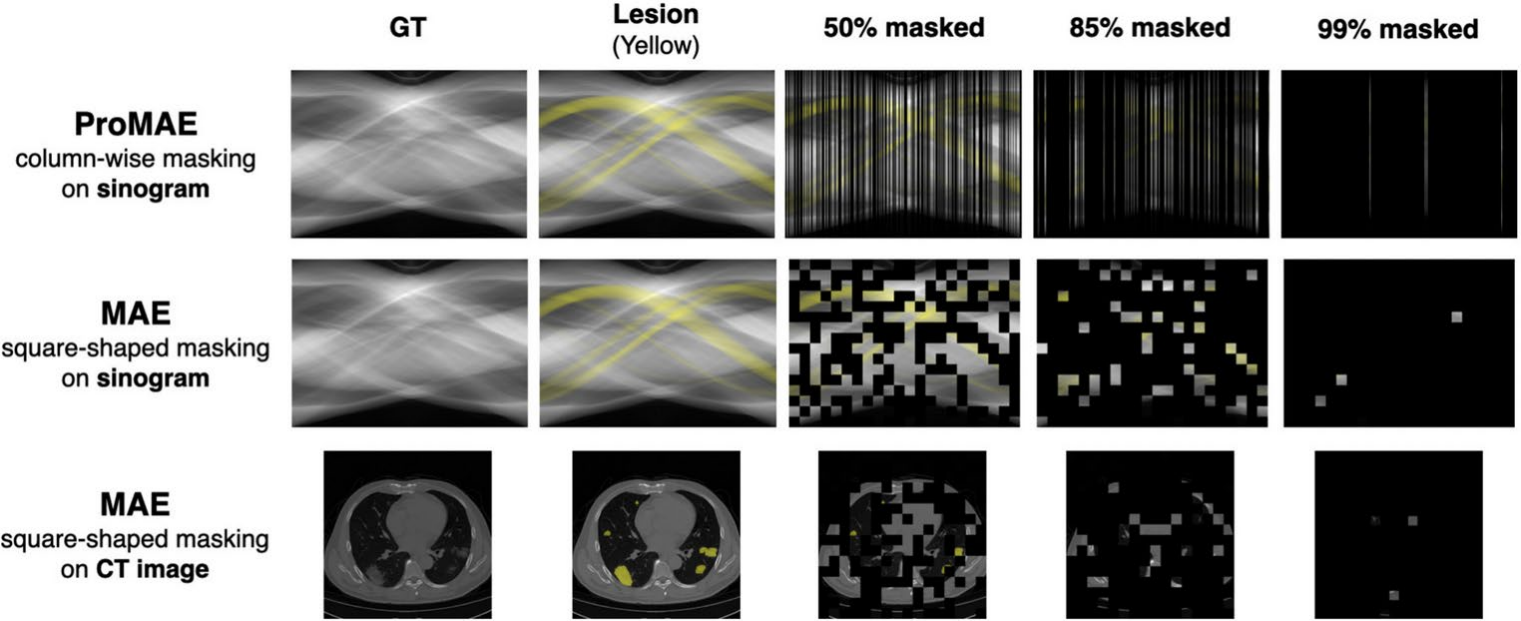
Masking strategy by model: The column-wise masking approach applied in ProMAE preserves lesion features (highlighted in yellow) better than the square-shaped masking used in MAE

Furthermore, column-wise masking naturally aligns with sparse-view scenarios where specific projection angles are masked. This approach directly incorporates the concept of sparse views into pre-training, as opposed to the square-shaped masking used in MAE, and helps retain lesion information even at high masking ratios. Figure 2 illustrates the application of square-shaped masking and column-wise masking on CT images and sinograms. In MAE’s square-shaped masking approach, as the masking ratio increases, lesion information (highlighted in yellow in Fig. 2) largely disappears. In contrast, ProMAE’s column-wise masking preserves lesion information even at very high masking ratios. This is because sinograms are likely to contain lesion details in every column, enabling effective extraction of lesion features even from a limited number of columns (projections).

In addition, by applying random masking, ProMAE can be trained to provide robust diagnostic results even in adverse situations where sampling is limited (for instance, due to errors in some samples or restrictions in imaging angles). While previous studies [47] have applied random column-wise masking on sinograms for various CT denoising tasks, ProMAE advances this approach by focusing on extracting features critical for disease classification.

The key advantages of column-wise tokenization and random masking are as follows.

**Fig. 3:**
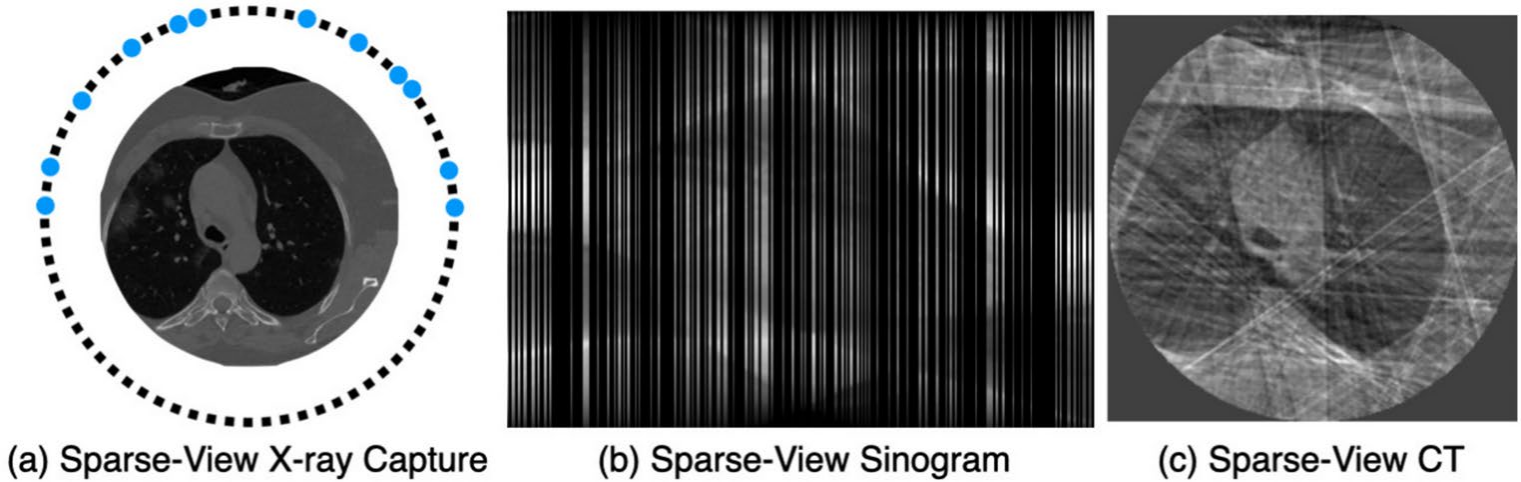
Randomly column-wise masking of the sinogram: The blue dots in (**a**) represent the points (angles) at which projections were acquired. These sparse projections form the sparse-view sinogram as shown in (**b**). This, in turn, is reconstructed to a low-quality CT image, as seen in (**c**), through Filtered Back Projection (FBP)

1. Restoring masked sinogram columns simplifies interpolation by leveraging the relationships between sine functions [21], making it more efficient than square-shaped masking of CT images. This enables stable learning even at high masking ratios.
2. Column-wise masking naturally aligns with sparse-view sinogram (Fig. 3b), where the masking ratio increases as the number of projections decreases. This masking approach preserves lesion information, enabling effective learning of lesion features required for classification from sparse-view sinograms with varying degrees of sparsity.
3. By applying random masking, the model is capable of processing projections sampled from arbitrary angles. This provides flexibility in imaging system design and enables robust diagnostic performance across diverse acquisition environments and outcomes.

In the experiments, we evaluated the performance of models employing various masking strategies. ProMAE uses column-wise masking, while MAE employs square token masking, and both methods were pre-trained with masking ratios of 50%, 75%, 85%, 95%, and 99%. The input data for ProMAE, the sinogram, consists of 360 columns (projections). Depending on the masking ratio, the masked sinogram contains 180, 90, 54, 18, or 4 X-ray projections, representing a sparse-view sinogram (Fig. 3b). When reconstructed, this corresponds to a sparse-view CT (Fig. 3c).

### Pre-training

**Fig. 4:**
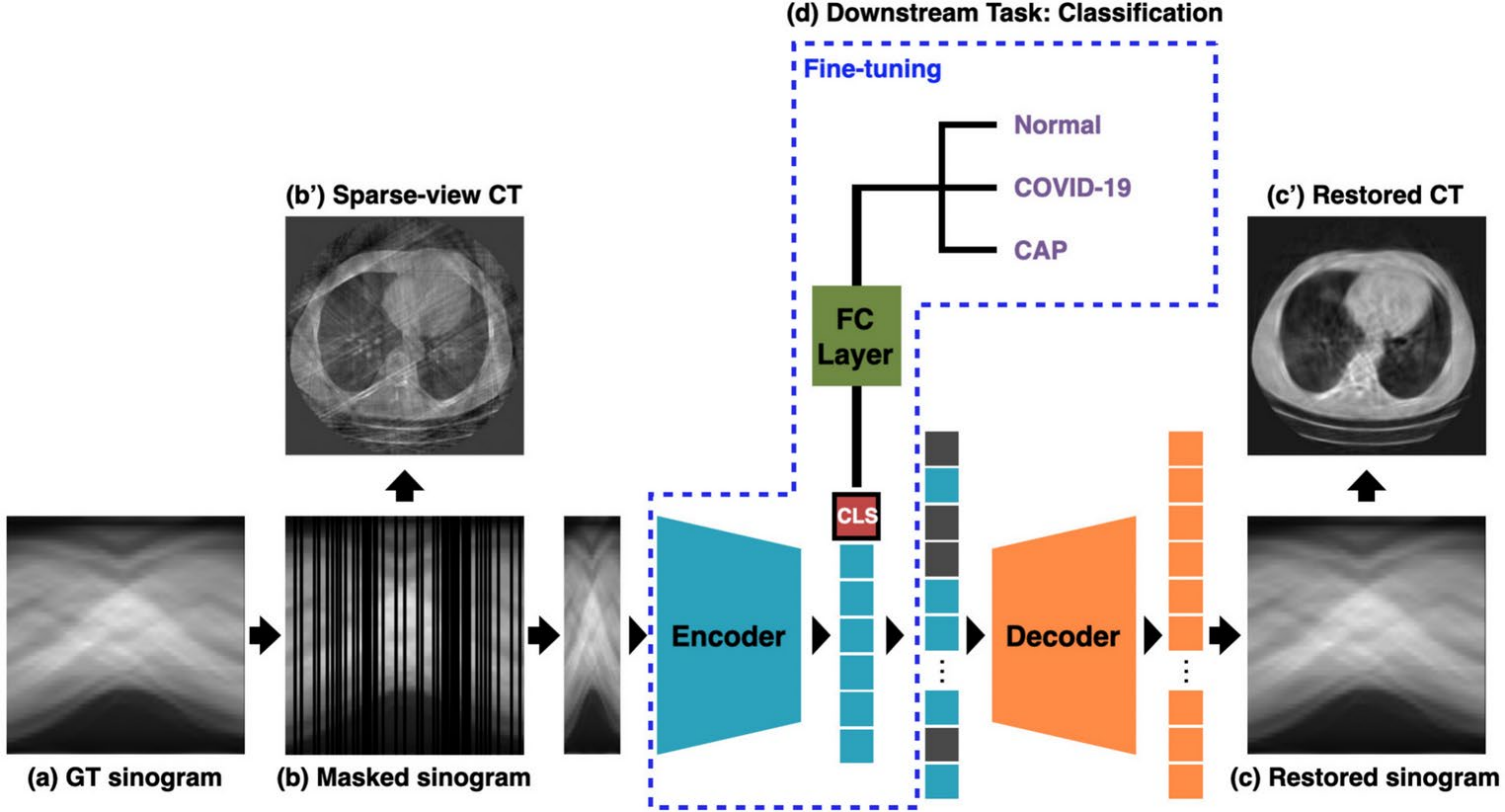
Overall Framework of ProMAE: ProMAE takes the GT sinogram (**a**) as input and performs pre-training using column-wise masking (**b**). The pre-training process aims to minimize the difference between the restored sinogram (**c**) and the GT sinogram (a) while encoding the sinogram features into a class token (CLS). Once pre-training is complete, the downstream task (**d**) involves classification learning using the class token. In the downstream task, both the fully connected (FC) layer and the encoder are fine-tuned, enabling comprehensive learning that enhances classification accuracy

The pre-training of ProMAE (Fig. 4**a**$\rightarrow$**b**$\rightarrow$**c**) closely follows the Masked Autoencoder approach, utilizing GT sinograms as input. After column-wise tokenization, $r\%$ of the tokens are masked. The class token (Fig. 4 CLS), which interacts with the unmasked (visible) tokens to learn the key features of the sinogram, is processed simultaneously by the ViT [10] encoder. The encoder outputs comprehensive representations that are passed to the decoder to restore the masked tokens. Restoration accuracy is measured using $L^2$ loss, aiming to align the full-view sinogram predicted from the sparse-view sinogram with the GT sinogram.

In ProMAE’s pre-training, various masking ratios (50%, 75%, 85%, 95% and 99%) are applied to determine the optimal ratio for extracting features best suited for the task, depending on the data. In fact, many transformer-based studies, including MAE, adjust the masking ratio during training according to the type of data and the difficulty of the task. For example, the language model BERT uses a 15% masking ratio [14], MAE for image data employs a 75% masking ratio [9], and video-MAE achieves optimal performance with a 90% masking ratio for video data [24]. Therefore, this study verifies which masking ratio is most effective in learning the characteristics of COVID-19 lesions during the restoration process.

Both the pre-trained ProMAE and MAE models are evaluated on the GT data validation set, and the model with the lowest validation loss is selected. To assess reconstruction quality, the restored sinogram is converted back into a CT image via Filtered Back Projection (FBP) (Fig. 4**c**$\rightarrow$**c**’) and compared to the GT CT image using PSNR and SSIM metrics.

To compare ProMAE and MAE, MAE pre-training was performed in two domains: CT and sinogram. While previous studies conducted MAE pre-training only on CT images [32], this study applied square-shaped masking on sinograms to compare restoration performance within the same domain as ProMAE. MAE pre-trained on CT images is denoted as MAE (CT), and MAE pre-trained on sinograms is denoted as MAE (sinogram). The differences between these methods are summarized in Table 2, and the training parameters are provided in Table 3.

**Table 2:** Comparison of Pre-training

Specs	ProMAE	MAE (CT)	MAE (sinogram)
Input (size)	GT sinogram ($256\times 360$)	GT CT ($256\times 256$)	GT sinogram ($256\times 368$)^1^
Number of tokens	360	256	368
Token shape	Column (256$\times$1)	Square (16$\times$16)	Square (16$\times$16)
Model size	51.0M	51.2M	51.3M

^1^ In MAE pre-training, the width of the GT sinogram was adjusted to 368 (a multiple of 16) to match the token shapes of CT and sinogram data

### Downstream task

The downstream task is a key stage of ProMAE, focusing on disease diagnosis using the class token extracted from the pre-trained encoder. An $r\%$ sparse-view sinogram is passed through the trained encoder to extract the class token, enabling classification based on the features of the sparse-view sinogram.

In the classification training conducted in the downstream task, a fully connected (FC) layer is applied to the 512-dimensional class token extracted from the sparse-view image to form a classifier, which predicts one of three labels: NonCOVID, COVID, or CAP. During this training process, both the FC layer and the encoder are fine-tuned simultaneously to achieve optimal performance. When a sparse-view image passes through the fine-tuned encoder, an enhanced class token is generated, and after processing through the trained FC layer, the diagnostic result is produced. Finally, the model with the highest accuracy on the validation dataset is selected as the final classifier.

**Fig. 5:**
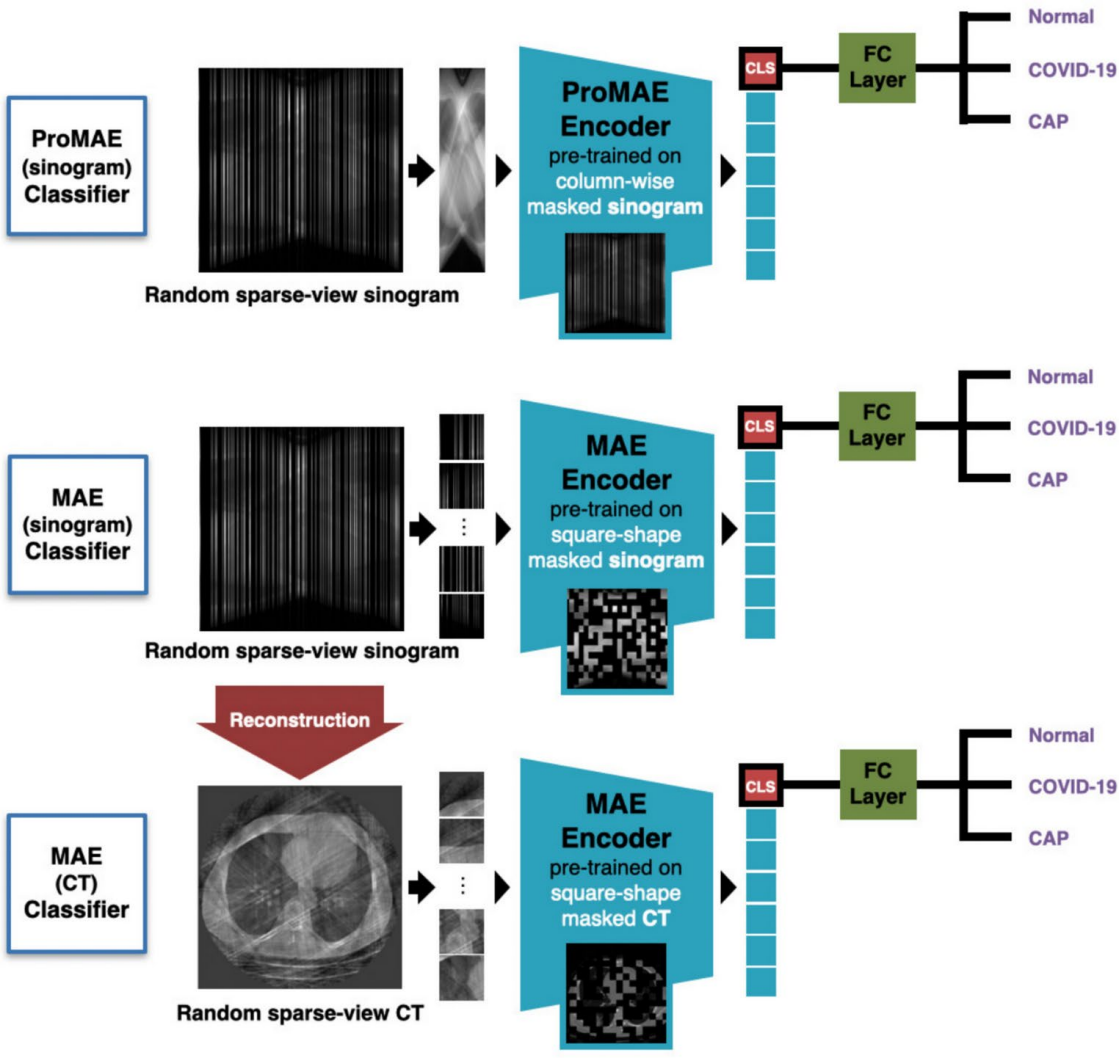
Classifiers of each model: The classifiers are trained in the downstream task following pre-training. The ProMAE classifier is trained to classify sparse-view sinograms after pre-training using random column-wise masking on GT sinograms. In contrast, the MAE classifier is trained to classify sparse-view data (sinograms or CT images) after pre-training on GT data using square-shaped masking

To evaluate the models, experiments were conducted using the fine-tuned classifiers to classify sparse-view images at sparsity levels of 50%, 75%, 85%, 95%, and 99%. In particular, to compare the performance of ProMAE and MAE, the following experiments were carried out (Fig. 5): ProMAE was used to classify sparse-view sinograms, and MAE (sinograms) was used to classify sparse-view sinograms. Additionally, MAE (CT) was used to classify sparse-view CT images, where the sparse-view CT images ($256\times 256$) were reconstructed from sparse-view sinograms ($256\times 360$).

The model was trained using an NVIDIA GeForce RTX 4090, and the training parameters are presented in Table 3.

**Table 3:** Training parameters

Parameters	Pre-training	Fine-tuning
Total epoch (warm UP)	3,000 (100)	50 (0)
Batch size	128	50
Weight decay	0.05	0.01
Learning rate	0.002	0.001
Embedded dimension	512	512
The number of heads	8	8
The number of layers	Encoder: 12, Decoder: 4	Encoder: 12, FC: 1

## Results

### Pre-training results

**Fig. 6:**
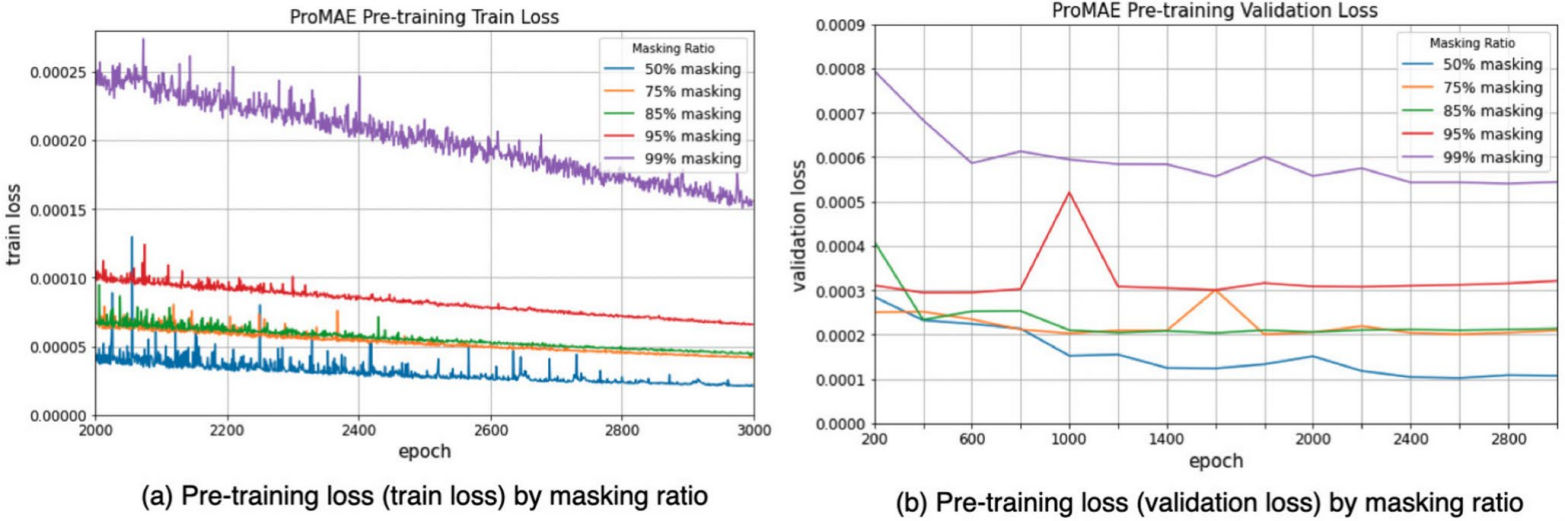
Train and validation loss of ProMAE pre-training: the pre-training loss of ProMAE increased for both the training and validation sets as the masking ratio increased

**Fig. 7:**
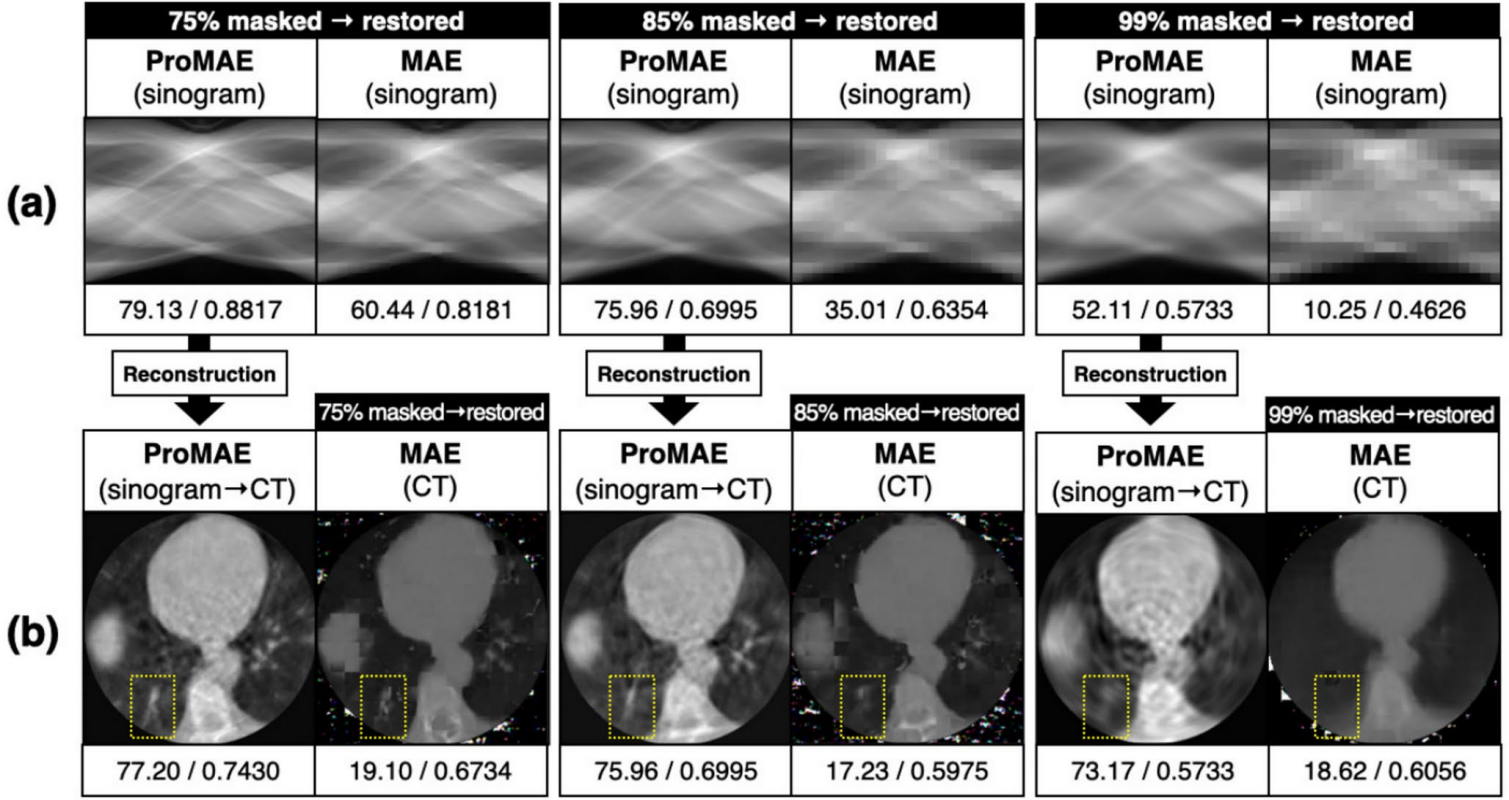
Pre-training Results: **a** A comparison of the restoration performance of masked sinograms between ProMAE and MAE. **b** A comparison of the CT images reconstructed from the sinogram restored by ProMAE with those restored by MAE. The yellow box indicates the lesion location, and the numbers at the bottom of each restored image represent the PSNR (left) and SSIM (right) of the restored image relative to the original image

ProMAE was pre-trained with masking ratios of 50%, 75%, 85%, 95%, and 99%. The encoder and decoder restore full-view sinograms from masked sinograms over 3000 epochs, and the model with the lowest validation loss is saved. Specifically, the best pre-trained models were selected at 2600 epochs for 50% masking, 1800 epochs for 75% masking, 1600 epochs for 85% masking, 400 epochs for 95% masking, and 2800 epochs for 99% masking. Figure 6 shows the loss trends, while Fig. 7 compares the pre-training results of ProMAE and MAE at sparsity levels of 75%, 85%, and 99%.

Figure 7a shows that ProMAE is able to restore sinograms effectively even under 99% masking, whereas MAE (sinograms) fails to properly restore sinograms at masking ratios above 85%. In fact, at all masking ratios, the PSNR and SSIM of the sinograms restored by ProMAE with respect to the GT sinogram are higher than those of the sinograms restored by MAE (sinograms). Figure 7b compares the CT images reconstructed by ProMAE and MAE (CT). For this comparison, the full-view sinogram restored by ProMAE was reconstructed into a CT image and then compared with the pre-training results of MAE (CT). Despite the high masking conditions, ProMAE successfully restores lesion locations (Fig. 7b highlighted by the yellow box), while MAE (CT) fails to reproduce them. This demonstrates that ProMAE can effectively extract lesion features by robustly restoring sinograms even under high sparsity conditions, with the extracted features being incorporated into the class token to enable high performance in subsequent downstream tasks.

### Downstream task performance

Table 4 summarizes the classification results of the fine-tuned classifiers for ProMAE and MAE. All experiments were repeated 5 times to evaluate classification accuracy. The best-performing model at each sparsity level is as follows: for GT data, MAE (CT) with 95% masking achieved an average accuracy of $0.984\pm 0.002$ (95% CI); for 50% sparse-view data, MAE (CT) with 95% masking achieved an average accuracy of $0.985\pm 0.002$ (95% CI); for 75% sparse-view data, MAE (CT) with 95% masking achieved an average accuracy of $0.979\pm 0.002$ (95% CI); for 85% sparse-view data, ProMAE with 99% masking achieved an average accuracy of $0.976\pm 0.005$ (95% CI); for 95% sparse-view data, ProMAE with 99% masking achieved an average accuracy of $0.976\pm 0.004$ (95% CI); and for 99% sparse-view data, ProMAE with 99% masking achieved an average accuracy of $0.959\pm 0.004$ (95% CI).

**Table 4:** Downstream task (Fine-tuning) results

Model (pre-training method)^1^	Accuracy performance^2^on sparse(%)-View					
	GT	50%	75%	85%	95%	99%
MAE (sinogram, 50% masking)	0.9596	0.8298	0.6957	0.6950	0.6770	0.6363
	(0.0022)	(0.0057)	(0.0085)	(0.0033)	(0.0034)	(0.0071)
MAE (sinogram, 75% masking)	0.9731	0.8573	0.7232	0.7097	0.6857	0.6498
	(0.0035)	(0.0067)	(0.0066)	(0.0136)	(0.0043)	(0.0037)
MAE (sinogram, 85% masking)	0.9658	0.8478	0.7025	0.7067	0.6851	0.6432
	(0.0012)	(0.0041)	(0.0091)	(0.0070)	(0.0041)	(0.0142)
MAE (sinogram, 95% masking)	0.9588	0.8424	0.7176	0.6927	0.6804	0.6322
	(0.0013)	(0.0068)	(0.0020)	(0.0049)	(0.0101)	(0.0032)
MAE (sinogram, 99% masking)	0.9613	0.8473	0.7151	0.7027	0.6507	0.6457
	(0.0041)	(0.0046)	(0.0056)	(0.0094)	(0.0075)	(0.0057)
MAE (CT, 50% masking)	0.9807	0.9782	0.9738	0.9659	0.9250	0.7584
	(0.0019)	(0.0030)	(0.0045)	(0.0019)	(0.0053)	(0.0045)
MAE (CT, 75% masking)	0.9804	0.9799	0.9709	0.9680	0.9307	0.7698
	(0.0026)	(0.0017)	(0.0023)	(0.0016)	(0.0029)	(0.0067)
MAE (CT, 85% masking)	0.9798	0.9771	0.9732	0.9672	0.9381	0.7791
	(0.0011)	(0.0017)	(0.0019)	(0.0024)	(0.0031)	(0.0075)
MAE (CT, 95% masking)	**0**.**9841**	**0**.**9850**	**0**.**9793**	0.9748	0.9489	0.8026
	(0.0012)	(0.0013)	(0.0017)	(0.0013)	(0.0016)	(0.0034)
MAE (CT, 99% masking)	0.9569	0.9469	0.9276	0.9037	0.8390	0.7127
	(0.0019)	(0.0061)	(0.0049)	(0.0046)	(0.0028)	(0.0038)
ProMAE (50% masking)	0.9654	0.9672	0.9679	0.9681	0.9675	0.8784
	(0.0023)	(0.0008)	(0.0008)	(0.0013)	(0.0011)	(0.0038)
ProMAE (75% masking)	0.9642	0.9681	0.9677	0.9691	0.9687	0.8597
	(0.0028)	(0.0012)	(0.0044)	(0.0011)	(0.0014)	(0.0035)
ProMAE (85% masking)	0.9642	0.9648	0.9658	0.9686	0.9695	0.9117
	(0.0030)	(0.0020)	(0.0011)	(0.0032)	(0.0014)	(0.0029)
ProMAE (95% masking)	0.9748	0.9741	0.9741	0.9734	0.9740	0.9417
	(0.0030)	(0.0022)	(0.0028)	(0.0006)	(0.0013)	(0.0018)
ProMAE (99% masking)	0.9740	0.9758	0.9762	**0**.**9756**	**0**.**9756**	**0**.**9586**
	(0.0021)	(0.0033)	(0.0018)	(0.0039)	(0.0029)	(0.0032)

Bold text represents the highest value

^1^ square-shaped masking for MAE, column-wise masking for ProMAE

^2^ average accuracy after 5 repeated measurements (standard deviation in parentheses)

For GT data, MAE (sinogram), MAE (CT), and ProMAE all demonstrated satisfactory performance with accuracies above 95%. However, MAE (sinogram) showed lower performance than both MAE (CT) and ProMAE at sparsity levels above 50%. In particular, the difference between the results of the two MAE (sinogram and CT) models, both trained with the same square-shaped masking strategy, indicates that square-shaped masking is more suitable for learning CT images than sinograms. ProMAE and MAE (CT) show similar performance at low sparsity levels, but as sparsity increases, the performance gap gradually widens. When comparing the best performance at each sparsity level, ProMAE achieves an accuracy of 95.86% at 99% sparsity, whereas MAE (CT) only reaches 80.26% at 99% sparsity, reflecting a difference of over 15%. This occurs because, in low-sparsity data, lesion shapes remain distinguishable, but in high-sparsity data, the lesion shapes become significantly degraded, making it difficult to extract lesion features. However, as shown in Fig. 2, sinograms with column-wise masking are able to preserve lesion information even at high sparsity levels, thereby maintaining high performance.

ProMAE demonstrates optimal performance across all sparsity levels when pretrained with 99% masked sinograms. This suggests that predicting 99% of the projection information from only 1% provides an ideal level of difficulty for the model to learn both the overall sinogram structure and subtle lesion characteristics. When pretrained in this way, ProMAE outperforms MAE (CT) on data with sparsity levels exceeding 85%, with the performance gap widening as sparsity increases. Conversely, for sparsity levels below 75%, MAE (CT) trained with a 95% masking ratio achieves higher performance. These results indicate that while MAE (CT) is effective in capturing lesion features from low-sparsity data, ProMAE’s training strategy becomes increasingly effective as data sparsity increases.

### Performance comparison with other models

**Table 5:** Comparision with other models

Model(domain)	Accuracy performance^1^ on sparse(%)-view						param^2^
	GT	50%	75%	85%	95%	99%	
ResNet-50^3^(CT)	0.9268	0.8926	0.8563	0.8346	0.7841	0.7079	25.6M
	(0.0063)	(0.0030)	(0.0059)	(0.0050)	(0.0077)	(0.0043)	
ConvNeXt-B^3^(CT)	0.8549	0.7905	0.7626	0.7352	0.7094	0.6631	87.6M
	(0.0071)	(0.0130)	(0.0094)	(0.0027)	(0.0060)	(0.0083)	
MAE^4^(sinogram)	0.9731	0.8573	0.7232	0.7097	0.6857	0.6498	51.3M
	(0.0035)	(0.0067)	(0.0066)	(0.0136)	(0.0043)	(0.0037)	
MAE^5^(CT)	**0**.**9841**	**0**.**9850**	**0**.**9793**	0.9748	0.9489	0.8026	51.2M
	(0.0012)	(0.0013)	(0.0017)	(0.0013)	(0.0016)	(0.0034)	
ProMAE^6^(sinogram)	0.9740	0.9758	0.9762	**0**.**9756**	**0**.**9756**	**0**.**9586**	51.0M
	(0.0021)	(0.0033)	(0.0018)	(0.0039)	(0.0029)	(0.0032)	

Bold text represents the highest value

^1^average accuracy after 5 repeated measurements (standard deviation in parentheses)

^2^parameter size

^3^test results after training on each sparse-view CT image

^4^pre-trained with 75% masking ratio. (Table 4, MAE (sinogram, 75% masked))

^5^pre-trained with 95% masking ratio. (Table 4, MAE (CT, 95% masked))

^6^pre-trained with 99% masking ratio. (Table 4, ProMAE (99% masked))

**Fig. 8:**
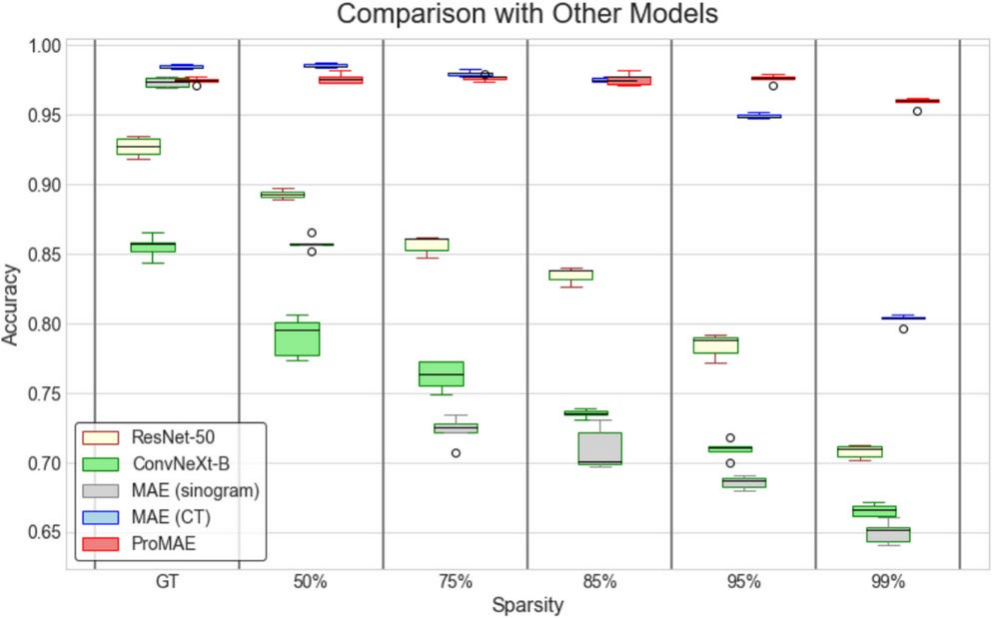
Comparision with other models: ProMAE demonstrates robust performance by maintaining over 95% accuracy across all sparsity levels, without significant performance degradation

Table 5 compares ResNet-50 [44], ConvNeXt-B [27], MAE, and ProMAE. ResNet-50 and ConvNeXt-B were trained and tested on sparse-view CT images, either by training on GT CT images and evaluating on GT CT images, or by training on $r\%$ sparse-view CT images and testing on $r\%$ sparse-view CT images. These two models were compared with MAE and ProMAE, which achieved the highest performance in Table 4.

MAE (sinogram) exhibited the lowest performance at sparsity levels above 75%. ConvNeXt-B, despite having the largest number of parameters, achieved only $0.855\pm 0.010$ (95% CI) accuracy even on GT data, indicating low efficiency. In contrast, ResNet-50, which was trained with the fewest parameters, could not match the classification performance of MAE (CT) and ProMAE on any sparse-view dataset, including GT data. Therefore, among the compared models, MAE (CT) performed better at sparsity levels of 75% or less, while ProMAE outperformed the other models at sparsity levels above 85%.

Figure 8 presents the results of 5 experiments for all models in the form of error bars. While models other than ProMAE exhibit a sharp performance drop at sparsity levels above 85%, ProMAE demonstrates robust performance across all sparsity levels.

## Conclusion and discussion

This study introduces ProMAE, a Masked Autoencoder-based model designed for COVID-19 diagnosis. ProMAE applies column-wise masking to sinograms, effectively learning diagnostic features even under extremely sparse-view conditions. Experiments on sparse-view data with sparsity levels of 50%, 75%, 85%, 95%, and 99% demonstrate that ProMAE outperforms ResNet, ConvNeXt, and MAE pre-trained on sinograms at all sparsity levels, and for sparsity levels above 85%, it even exhibits superior performance compared to MAE pre-trained on CT images, thereby proving its robustness under high sparsity conditions.

ProMAE processes sinograms directly, eliminating the need for CT image reconstruction and simulating random sparse-view scenarios. This reduces the dependency on dense projection data and high-performance hardware. Such an approach enables the development of lightweight devices that can diagnose using only a minimal number of projections from random angles, thereby minimizing cumulative radiation exposure for operators while significantly enhancing diagnostic efficiency and accessibility. In large-scale outbreaks like COVID-19, ProMAE can deliver diagnostic results within seconds on low-performance hardware, easing the burden on healthcare systems managing large patient volumes and facilitating rapid initial diagnosis even in resource-limited settings.

In conclusion, ProMAE represents a significant advancement in sparse-view image analysis and COVID-19 diagnosis. Future research should aim to optimize the masking ratios for various lesion types not only in COVID-19 and pneumonia but also in a range of pulmonary diseases. Moreover, its application should be extended to other body regions where CT diagnosis is crucial, such as in brain and abdominal disorders, to further enhance its utility.
